# Sexual and reproductive health and rights: still ground zero for UHC in the WHO European Region

**DOI:** 10.1080/26410397.2020.1841379

**Published:** 2020-11-25

**Authors:** Nino Berdzuli, Mikael Ostergren, Ketevan Chkhatarashvili, Martin W. Weber, Susanne Carai

**Affiliations:** a Director, Division of Country Health Programmes, World Health Organization Regional Office for Europe, Copenhagen, Denmark; b Consultant, World Health Organization Regional Office for Europe, Copenhagen, Denmark; d Programme Manager, Child and Adolescent Health, World Health Organization Regional Office for Europe, Copenhagen, Denmark; e Consultant, World Health Organization Regional Office for Europe, Copenhagen, Denmark; Ph.D student, University Witten Herdecke Faculty of Medicine, Witten, Germany

**Keywords:** sexual and reproductive health, universal health coverage, health benefit package, primary health care, quality of health care

Sexual and reproductive health and rights (SRHR) is a litmus test for progress towards universal health coverage.^1^ It requires a well-functioning health system, but in addition policy and legislative barriers need to be addressed and human rights and gender equality respected and promoted. Furthermore, many sexual and reproductive health (SRH) interventions, such as prevention of and services for victims of violence against women, as well as access to comprehensive sexuality education, require multi-sectoral action and policies beyond the health sector. Recently, the COVID-19 pandemic has put a strain on healthcare systems, even the most robust, challenging the provision of essential health services, including for SRH. Notwithstanding the current need to focus on COVID-19 response, SRHR becomes more vital than ever to ensure continued access to these services and that hard-won progress in SRHR is not set back. Achievements and challenges in SRH are an indicator of how well UHC is progressing, and its prospects of success. In addition, advancing programmes that improve women’s lives and rights is essential to sustaining civil society support for the UHC agenda. Thus, SRHR is at the core and the vanguard of UHC and it cannot be achieved without a strong focus on these services.

## An assessment of SRHR in selected countries of the European region

But how well is SRHR integrated in the UHC agenda in the WHO European region? To answer that, assessments of sexual, reproductive, maternal, newborn, child and adolescent health (SRMNCAH) in the context of UHC were carried out by the WHO European Office in collaboration with the respective Ministries of Health in Albania, Azerbaijan, Kazakhstan, Kyrgyzstan, the Republic of Moldova and Romania.^2^ The assessments were conducted to determine which SRMNCAH services were included in policies related to UHC; to determine the extent to which, and at what cost, they were available to populations that they were intended for; to ascertain – via a tracer methodology and equity lens – barriers within health systems regarding provision of SRMNCAH services; and to distinguish priorities for action and develop policy recommendations. The protocol that was developed for the assessments involved both a document review and a subsequent country visit. For each country visit, interviews were conducted with policy-makers, representatives of the government and health insurance fund, health facility managers, doctors, midwives, nurses, patients, clients and partners. The interviews were conducted using semi-structured questionnaires. In addition, primary-, secondary- and tertiary-level healthcare facilities were visited.

Three of the six tracer interventions examined related to SRHR: antenatal care, with a focus on pre-eclampsia; sexually transmitted infections (STIs) excluding HIV; and adolescent-friendly health services, with a focus on SRH. The others were transport of sick neonates; and case management of common childhood conditions, with a focus on pneumonia and immunisation.

### Health systems barriers to the provision of and access to SRHR services

The existing official state health benefit packages were often under-funded and this lack of resources led to rationing and out-of-pocket (OOP) payments,^3^ including for SRH. It was not possible to track expenditure for specific SRH services but, overall, high OOP payments were a challenge in all the countries, varying from 21% to 79% of total health spending, with OOP being almost 50% or more in four of the countries. In addition, there were important gaps in SRH services included in the health benefit packages. With the exception of maternal health, other SRH services were not explicitly recognised or included in the benefit packages. For example, contraceptives were not fully included in the health benefit packages in many countries, and emergency contraception and HPV vaccination were not included in any. Also absent were abortion services, other than for miscarriage or medical reasons. The availability of these services was limited mainly to hospital settings in urban areas. Cervical cancer screening was not included in the health benefit packages in Azerbaijan and Kyrgyzstan.

There were no clearly defined criteria or processes in any of the countries for determining which services and medicines to subsidise or include in the health benefit packages. Instruments for applying criteria, such as Health Technology Assessments (HTA), were seldom used. Some interventions were covered by vertical programmes, whereas others were included in the health benefit packages of insurance schemes, which caused an additional fragmentation in the overall health system. In addition, clients and health service providers were often not informed or fully aware of the entitlements within the benefit package.

The Primary Health Care (PHC) services for SRH were often found to be fragmented, in line with previous studies.^4^ Family doctors were not always skilled and/or provided with capacity to deliver essential SRH services – for example simple STI treatment and IUD insertion – which led to multiple referrals. Nurses and midwives, who could strengthen family doctors’ ability to deliver services, were underused. In addition, there were shortages in the health workforce at the PHC level. All countries viewed the recruitment and retention of health workers as a challenge, especially in rural areas. The reported reasons for this were low salaries and poor working conditions. Finally, the PHC level was often funded on a per capita basis which, compared with the fee-for-service and case-based payments used for specialist and hospital care in many of the countries, is a low-powered incentive. All this led, in many instances, to a lack of trust in and bypassing of the PHC level by clients.

### Multisectoral approaches to ensure SRHR for all

Data on violence against women was limited and likely to be under-reported in all the assessed countries according to a complementary document review conducted in the context of the assessments.^5–7^Acts of violence were prosecuted only when a victim lodged a complaint and, in many cases, women did not want to report the act and press charges. In all countries, services were limited for those who had experienced gender-based violence. Healthcare workers were not always trained and able to recognise signs of violence in both women and children, and NGOs provided the majority of support and shelters. Three countries (Albania, the Republic of Moldova and Romania), though, had developed national strategies to prevent and address violence against women, together with action plans, and a pilot action plan has been established in a region of Kazakhstan.

Only in Albania, which had the lowest adolescent birth rate, was sexuality education in schools mandatory. Sexuality education was either not part of the curriculum or was optional – dependent on individual teachers – in the other countries that were included in the assessment. Pilot projects on sexuality education were, however, initiated and ongoing in some countries.

### State of SRHR as reflected in three tracer interventions

All countries except Azerbaijan and Romania had adopted as policies the WHO recommendations on antenatal care (including a minimum of eight contacts during pregnancy). Furthermore, population coverage was high for most countries (76–95%). However, there were challenges in all countries in terms of antenatal care service quality. Services were often fragmented, and pregnant women experienced multiple referrals, needing to see many different providers. There was a lack of confidence in the detection and management of complications such as pre-eclampsia in cases in which family doctors were the main providers of antenatal care.

The organisation of services to manage STIs was also often fragmented, again involving multiple referrals and services not fully available at the primary health care (PHC) level. STI testing was purportedly free in all countries, with syphilis testing included in the antenatal care package. However, patients themselves frequently paid – fully or partially – for treatment. Patients often by-passed family doctors and went directly to specialists (venereologists and dermatologists), often in the private sector, or at tertiary care level. Reasons included lack of laboratory services for STI diagnosis at PHC level, confidentiality issues and perceived better treatment at specialist/tertiary level.

Clear policies and interventions focusing on adolescent SRH were lacking in most countries. Adolescents younger than 18 years of age required parental consent to access services under protocols and legislation in the majority of countries assessed. Even where legislation allowed younger adolescents to access services in principle, health workers were not always aware of the legislation or were not always comfortable providing SRH services for them for other reasons. The scope of services that were provided often entailed only health information or health promotion; services such as contraception and STI treatment were rarely accessible to adolescents. A major barrier reported by care-seeking adolescents was a lack of confidentiality. This was especially the case when the services were provided at the local, PHC level.

## Lessons learned and the way forward

The assessments showed that many of the challenges for SRHR in the context of UHC are rooted in broader health systems issues. The overall capacity for strategic purchasing of health services needs strengthening in order to respond to the SRH needs and to deliver SRH services most efficiently, particularly at the PHC level.

Among the tracer interventions, “non-controversial” interventions, such as ANC, performed better than adolescent SRH services that met legislative, policy and societal barriers precluding adolescents under 18 years accessing services without parental consent. Even when policies were in place, health workers’ attitudes towards adolescent SRH and stigmatisation of people with STIs at times formed barriers to access to comprehensive services.

The lack of criteria and processes for designing the health benefit packages may negatively affect the inclusion of SRHR. Implicit choices may reflect societal and political norms rather than criteria such as health and efficiency, equity, targeting of vulnerable populations, financial protection and rights. SRHR services such as adolescent SRH, contraception, abortion care and STI treatment risk being excluded if explicit criteria are not set and gains in SRHR may be reversed. In addition, even when services are included in the health benefit package, they may not be provided due to under-funding and the fact that health benefit packages at times are political “wish-lists” rather than based on available resources and population needs.

UHC is still considered mainly a question for the health sector and delivery of services. SRHR interventions requiring multi-sectoral action beyond health services, such as prevention of and services for victims of violence against women and access to comprehensive sexuality education, are not fully included in the UHC agenda.

The assessments sparked a policy dialogue with multiple stakeholders in each country, including with decision-makers of health insurance funds. The policy dialogue took place directly in connection with the assessments, during a multi-country meeting with all the assessed countries and through follow-up with each country. As a result, the development of SRH action plans was initiated in two countries with a view to inclusion of SRH interventions in the health benefit packages. In a third country, amendments on reproductive health and rights were made to a new “Code on Populations and Health Systems” and access to SRH services for teenagers under 16 years of age without parental consent was being introduced. Overall, the assessments presented an opportunity to build and strengthen the evidence base for UHC and SRHR to more effectively support policy change for SRHR.

After the assessments in the six countries, an additional three countries (Greece, Tajikistan and Uzbekistan) have done similar assessments. As the integration of SRHR into the UHC agenda is a continuous process, countries in the WHO European region are encouraged to carry out similar reviews with an aim to track SRHR in advancing UHC and as an instrument for policy dialogue around it.

Future assessments would also need to take into account the lessons learned from the COVID-19 pandemic. SRH interventions such as family planning and contraception were among the most frequently disrupted areas reported in a recent pulse survey, conducted by WHO, in 105 countries.^8^ This provides an opportunity to re-think and review service delivery strategies for SRH. During the COVID-19 pandemic, the world has experienced an unprecedented demand on individuals to play a greater role in protecting their own health. In addition to the COVID-19 specific self-care measures such as physical distancing, good respiratory hygiene and hand washing, it applies to many other areas, especially for SRH in antenatal care, childbirth, family planning, safe abortion, sexually transmitted infections and sexual health.^9^

Finally, the European Programme of Work, 2020–2025 – “United action for better health in Europe” (EPW)^10^ of the WHO Regional Office for Health sets priorities for the coming five years. With SRH as a key priority in moving towards UHC, its implementation, if sufficiently resourced, may provide a strong framework for change and promoting better SRHR in the region.

## Disclaimer

The authors’ views expressed in this article are theirs and do not necessarily reflect the policies and positions of the World Health Organization.
